# Childhood Obesity May Be Linked to Feeding Habits and Screen Time

**DOI:** 10.7759/cureus.50933

**Published:** 2023-12-22

**Authors:** Ali Atwah, Emad Koshak, Maher S Shalabi, Abdulrahman Alsulami, Ahmed S Alsaedi, Osama Alharbi, Ziyad Almalki, Ahmed Moamina

**Affiliations:** 1 Pediatrics, King Abdulaziz University Faculty of Medicine, Rabigh, SAU; 2 Internal Medicine, King Abdulaziz University Hospital, Jeddah, SAU; 3 Pediatrics, University of Jeddah, Jeddah, SAU; 4 Medicine, King Abdulaziz University Faculty of Medicine, Rabigh, SAU

**Keywords:** risk factors for obesity, saudi arabia, cow’s milk, breastfeeding, obesity, overweight, childhood

## Abstract

Background

Childhood obesity is an alarming health problem. Early feeding habits and factors are among the etiological factors contributing to obesity.

Objectives

The objective of this study is to evaluate the correlation between breastfeeding, alongside other relevant factors, and their potential role as preventative measures against obesity.

Methods

A cross-sectional hospital-based study was conducted on children who attended a pediatric clinic. Demographic, clinical, and anthropometric measurements were taken from the hospital records. A questionnaire was completed by parents telephonically. Overweight was identified as a body mass index (BMI) of > 85-95% and obesity as a BMI of > 95%.

Results

A total of 101 children, with a mean age of 8.88 ± 4.01 (range one to 18) years, were involved, of whom 58.4% were boys. A high BMI (overweight or obese) was found in 30 (29.7%) children. The highest BMIs were among soft drink consumers [two children (66.7%) consumed daily and eight children (40%) consumed monthly], high birth weight in two children (40%), cow’s milk formula feeding in eight children (38%), and weekly fast food consumption in 18 children (35%), none of these were statistically significant. Nevertheless, there was a significant association between mean electronic device usage and high (204.5 ± 164.76 hours) and normal BMI (147.61 ± 110.24 hours) (p-value < 0.05).

Conclusion

This small cross-sectional study shows that almost one-third of the included children were overweight or obese, which is comparable to what has been published in the literature. Moreover, there was a potential link between some factors and obesity, especially screen time, which may contribute to the controversial literature.

## Introduction

Obesity and overweight during childhood are two of the most concerning health issues today [1,2]. Obesity in children is defined as a body mass index (BMI) of the 95th percentile or above and overweight as a BMI greater than the 85th percentile and less than the 95th percentile on the BMI-for-age growth chart [3]. The complications of obesity in childhood are multiple, including reflux disease, fatty liver, early development of type 2 diabetes, cardiovascular complications, psychiatric disorders, and musculoskeletal diseases [4].

The occurrence of obesity in children has increased worldwide, and there is an epidemic of obesity in the pediatric age group [5]. Globally, 39 million children under the age of five years are overweight or obese, and more than 340 million children aged between five and 19 are overweight or obese [6]. The prevalence of obesity is consistently increasing in developed and developing countries [7]. According to the WHO, 30% of Saudi children and adolescents are obese or overweight [8]. A Saudi study has shown that there has been a significant increase in the prevalence of obesity, with 13.4% of children being overweight and 18.2% of adolescents being obese. This represents a doubling in the prevalence over 10 years [9].

Childhood obesity is a multifaceted issue with various reported contributing factors, including family history and genetics, dietary habits, physical activity levels, and sleep patterns [2]. Children with a family history of obesity are more likely to struggle with weight issues, and frequent consumption of high-calorie, nutrient-poor foods exacerbates this issue [10]. A sedentary lifestyle and lack of regular physical activity further contribute to obesity, which makes it essential to promote healthy eating habits, encourage physical activity, and address sleep habits for adequate rest.

Moreover, infancy has a crucial effect on children's health because of the accelerated brain and body development during this period. Therefore, it is an important period in the prevention of obesity and its accompanying complications [11]. Breastfeeding has been linked to the provision of some protection against several illnesses, including obesity, and its absence is one of the risks linked with childhood obesity [12,13]. Most national pediatric and many international societies emphasize the recommendation to start breastfeeding within the first hour of life, followed by exclusive breastfeeding during the first six months, then adding complementary foods and continuing breastfeeding for the first two years of life [14-17]. Furthermore, some organizations recommend limiting screen time for children - no screen time for children younger than two years, no more than one hour per day for children aged two to five years, and consistent limits for older children [18]. In addition, they recommended limiting the consumption of soft drinks and reducing the consumption of fast food to once per week [19].

A comprehensive understanding of the various risk factors that may lead to childhood obesity is required to implement effective prevention strategies. Hence, this study aimed to assess the relationship between early and late feeding habits and other factors and obesity in our society.

## Materials and methods

Study design and setting

A retrospective cross-sectional population-based study was conducted to examine the relationship between breastfeeding and the obesity rate. Electronic medical records of children (from both genders), aged between one and 18 years, who attended the pediatric clinic at King Abdulaziz University Hospital (KAUH), were reviewed. The Ethical Research Committee at King Abdulaziz University’s Faculty of Medicine approved our research proposal (reference number 207-22), ensuring that the research study has been reviewed and meets the required ethical standards.

Verbal consent was obtained telephonically from the children’s parents. The data collected was divided into two divisions: firstly from hospital records review which provided data related to the child's information in the medical record, including age, gender, nationality, weight, height, BMI, and birth weight. The other division was parental surveys completed telephonically by the children’s parents and included questions regarding the type of feeding during the first six months [exclusive breastfeeding, cow’s milk formula (CMF), or a mix], duration of breastfeeding, number of minutes spent on electronic devices daily, fast food consumption, soft drink consumption, maternal smoking during pregnancy, and daily sleeping hours.

The children’s anthropometric measurements were taken by registered expert pediatric nursing staff. The children's weight and height were measured using standardized protocols. Weight was measured to the nearest 100 grams on an electronic scale, while height was measured with a wall-mounted stadiometer to the nearest 0.1 centimeter. Per the nurses' reports, the measurements of the children's weight were taken while the children were dressed in light clothing and without shoes. The weight of each child in kilograms was divided by the square of their height in meters to calculate the BMI, which was plotted in the growth chart of the Saudi Ministry of Health (MOH).

Inclusion criteria for the study were defined as children aged between one to 18 years (according to Saudi Arabia's children classification), no chronic diseases, and attendance at a pediatric clinic. Exclusion criteria were defined as children with chronic diseases (DM, celiac, etc.) and missing data or records.

Sample size

To possess sufficient statistical power to detect meaningful associations, we determined the necessary sample size for our research endeavor. Aiming for a power of 0.80, a standard alpha level of 0.05, and an anticipated effect size (Cohen's d) of 0.5, which is regarded as moderate and commonplace in social science research, we projected a total sample size of 126 participants to support a comparative analysis between two groups. G*Power (Heinrich-Heine-University Düsseldorf, Düsseldorf, Germany) was employed to calculate the sample size.

Statistical analysis

The collected data were analyzed using SPSS software (IBM Corp. Released 2012. IBM SPSS Statistics for Windows, Version 21.0. Armonk, NY: IBM Corp). Quantitative and qualitative data were summarized using descriptive statistics. Percentages were used to express qualitative variables, while mean and standard deviations were used for quantitative variables. Statistical analyses were performed to ascertain the significance of the independent variables and the correlations between those and the dependent variables. This study used Fisher’s exact tests to examine the relationship between the categorical data and the BMI (normal versus high), due to the small sample size, while independent t-tests were used to compare the mean for the numeric data. A p-value < 0.05 was considered statistically significant.

## Results

A total of 500 hospital records of children were identified. Only 101 children were involved in the study because of the low response rate to phone communication. Their mean age was 8.88 ± 4.01 (ranging from two to 18 years). The children were divided into three categories: 40 preschool children (39.6%), 33 school children (32.7%), and 28 adolescents (27.7%). Fifty-nine were males (58.4%), and 42 were females (41.6%). Regarding nationality, the number of Saudis was 76 (75.2%), and the number of non-Saudis was 25 (24.8%).

Forty-one (40.6%) of those children were exclusively breastfed until the age of six months, 21 (20.8%) were on full formula feeding, and 39 (38.6%) were on both breastfeeding and formula feeding. Regarding the birth weight of the studied cases, the mean birth weight was 2.82 ± 0.75 (ranging from one to five) kilograms, 69 of the children were born with normal birth weight (68.3%), 27 were born with low birth weight (26.7%), and only three were born with high birth weight (5%). In addition, 93 (92%) mothers did not smoke during their pregnancy, while eight (7.9%) mothers did smoke during pregnancy.

Of the recruited children, 68 (67.3%) had never consumed soft drinks, while three (3%), 15 (14.9%), and another 15 (14.9%) consumed soft drinks on a daily, weekly, and monthly basis, respectively. Moreover, 29 (28.7%) had never eaten fast food, while six (5.9%), 51 (50.5%), and 14 (14.9%) had eaten fast food on a daily, weekly, and monthly basis, respectively. The mean hours of sleep were 9.05 ± 2.3 (ranging from 5-17), while 80 (79.2%) had been going to bed before 9 p.m. and 21 (20.8%) after 9 p.m. The mean screen time (TV and electronic devices) was 164.5 ± 130.6 (ranging from 0-60) minutes.

The mean BMI of the studied cases was 18.02 ± 4.84 (ranging from 12.3 to 32.8). The majority, 71 (70.3%) children, had a normal BMI. Thirty (29.7%) of the studied children had a high BMI, with 12 (11.9%) being overweight and 18 (17.8%) obese. All socio-demographic data are demonstrated in Table 1.

**Table 1:** Participants socio-demographic characteristics BMI: body mass index

Variable	Category	Frequency	Percent
Gender	Female	42	41٫6
	Male	59	58٫4
Age in years	Mean ± SD (range)	8.88 ± 4.01 (2 - 18)	
Age-categorization	Early Childhood	40	39٫6
	School children	33	32٫7
	Adolescents	28	27٫7
Nationality	Non- Saudi	25	24٫8
	Saudi	76	75٫2
Type of breastfeeding	Both	39	38٫6
	Fully breastfeeding	41	40٫6
	Fully formula feeding	21	20٫8
Birth weight	Mean ± SD (range)	2.82 ± 0.75 (1 – 5)	
Birth weight categories	Low birth weight (<2.5)	27	26.7%
	Normal birth weight (2.5-4 Kg)	69	68.3%
	High birth weight ( >4 Kg)	5	5%
Mother smoke	No	93	92٫1
	Yes	8	7٫9
Soft-drink consumption	Never	68	67٫3
	Daily	3	3٫0
	Weekly	15	14٫9
	Monthly	15	14٫9
Fast-Food Consumption	Never	29	28٫7
	Daily	6	5٫9
	Weekly	51	50٫5
	Monthly	14	14٫9
Sleeping before 9 p.m.	After	80	79٫2
	Before	21	20٫8
Hours of sleep/day	Mean ± SD (range)	9.05 ± 2.3 (5 – 17)	
Electronic devices use (minutes/day)	Mean ± SD (range	164.5± 130.6 (0 – 660)	
Child BMI	Mean ± SD (range)	18.02 ± 4.84 (12.3– 32.8)	
BMI category	Normal	71	70٫3
	High BMI (overweight & obesity)	30	29٫7
	Overweight	12	11٫9
	Obesity	18	17٫8

The following correlations were found between the different factors and a higher BMI. Eleven (26.2%) females and 19 (32.2%) males had high BMIs. Nine and 14 of the Saudis were overweight and obese, respectively, while three and four of the non-Saudis were overweight and obese, respectively. The children who had high BMI's were classified according to age as follows: 11 (27.5%) preschoolers, 10 (31.3%) school-age children, and nine (31%) adolescents.

The following correlations were found between early feeding patterns and higher BMIs. The highest prevalence rate of high BMI was among children who had been on cow’s milk formula, eight (38%), followed by children who had exclusive breastfeeding, 15 (36.6%), and the lowest prevalence was among the seven (18%) children who had been on both types of feeding (mixed). One child with a high birth weight was overweight, and one was obese, compared to 22 children with a normal birth weight who had a high BMI, and six (22.2%) children with a low birth weight who had a high BMI. None of the children whose mothers smoked during pregnancy were obese, and two of them were overweight (Table 2).

**Table 2:** Difference between body mass index (BMI) among different feeding habits and cofactors

		BMI category								p-value
		Normal		Overweight		Obesity		High BMI		
		N	%	N	%	N	%	N	%	
Gender	Male	40	67.8%	6	10.2%	13	22%	19	32.2%	0.625
	Female	31	73.8%	6	14.3%	5	11.9%	11	26.2%	
Age-categorization	Early childhood	29	72.5%	3	7.5%	8	20%	11	27.5%	0.925
	School children	22	68.7%	3	9.4%	7	21.9	10	31.3%	
	Adolescents	20	69%	6	20.7%	3	10.3%	9	31%	
Nationality	Non-Saudi	18	72%	3	12%	4	16%	7	28%	1
	Saudi	53	69.7%	9	11.8%	14	18.5%	23	30.3%	
Type of breastfeeding	Both	32	82.1%	3	7.7%	4	10.3%	7	18%	0.121
	Fully breastfeeding	26	63.4%	5	12.2%	10	24.4%	15	36.6%	
	Fully formula feeding	13	61.9%	4	19%	4	19%	8	38%	
Birth weight	Mean ± SD (range)	2.762		2.8125		3.061		2.961		0.226
Birth weight categories	Low	9	33.3%	4	14.8%	2	7.4%	6	22.2%	0.467
	Normal	17	24.6%	7	10.1%	15	21.7%	22	31.8%	
	High	2	40%	1	20%	1	20%	2	40%	
Mother smoke	No	65	69.95	10	10.8%	18	19.45%	28	30.1%	0.558
	Yes	6	75%	2	25%	0	0%	2	25%	
Soft drink consumption	Never	49	72.1%	7	10.3%	12	17.6%	19	27.9%	0.331
	Daily	1	33.3%	0	0%	2	66.7%	2	66.7%	
	Weekly	12	80%	2	13.3%	1	6.7%	3	20%	
	Monthly	9	60%	3	20%	3	20%	6	40%	
Fast food consumption	Never	22	75.9%	1	3.4%	6	20.7%	7	24.1%	0.691
	Daily	5	83.3%	0	0%	1	16.7%	1	16.7%	
	Weekly	33	64.7%	8	15.7%	10	19.6%	18	35.3%	
	Monthly	11	73.3%	3	20%	1	6.7%	4	26.7%	
Sleep before 9 p.m.	After	56	70%	9	11.3%	15	18.8%	24	30.1%	1
	Before	15	71.4%	3	14.3%	3	14.3%	6	28.6%	
Hours sleep/day	Mean ± SD (range)	9.03± 2.07		9.75± 4.27		8.67±1.53		9.1±2.93		0.464
Electronic devices use (minutes/day)	Mean ± SD (range)	147.61±110.24		205.42±197,23		203.89±145.41		204.5±164.76		0.045

Weekly fast food consumption was associated with the highest prevalence rate (18, 21.4%) of high BMI, followed by monthly fast food consumption (4, 26.7%), no fast food consumption (7, 24.1%), and daily fast food consumption (1, 16.7%). The highest prevalence of high BMI was among children who consumed soft drinks daily, followed by monthly, never, and weekly, and the percentages were 66.7%, 40%, 24.1%, and 20%, respectively. The prevalence of high BMI was almost similar among children who went to bed before and those who went to bed after 9 p.m., which was 30.1% and 28.6%, respectively (Table 2). None of the factors examined demonstrated statistical significance in their correlation with BMI categorization. The analysis subdivided the factors into three distinct age categories. It revealed a positive correlation between male gender and high BMI in school-age children (p < 0.05), but this correlation was not observed in the other two age groups. Similarly, there was a positive correlation between Saudi nationality and high BMI in school-age children (p < 0.05) but not in the other groups. Regarding other examined factors, such as the type of breastfeeding, fast food consumption, soft drinks consumption, and sleep time, the study found no statistically significant correlations with BMI across any age group (Table 3).

**Table 3:** Associations between cofactors and lifestyle behaviors and BMI categorization among the three age groups BMI: body mass index

			BMI				p-value
			Normal		High BMI		
Gender	Early childhood	F	10	62.5%	6	37.5%	0.247
		M	19	79.2%	5	20.8%	
	School children	F	14	87.5%	2	12.5%	0.012
		M	8	47.1%	9	52.9%	
	Adolescents	F	7	70%	3	30%	0.901
		M	13	72.2%	5	27.8%	
Nationality	Early childhood	Non-Saudi	7	70%	3	30%	0.838
		Saudi	22	73.3%	8	26.7%	
	School children	Non-Saudi	7	100%	0	0%	0.035
		Saudi	15	57.7%	11	42.3%	
	Adolescents	Non-Saudi	4	50%	4	50%	0.112
		Saudi	16	80%	4	20%	
Type of breastfeeding	Early childhood	Both	12	75%	4	25%	0.958
		Fully breastfeeding	12	70.6%	5	29.4%	
		Fully formula feeding	5	71.4%	2	28.6%	
	School children	Both	9	90%	1	10%	0.169
		Fully breastfeeding	6	54.5%	5	45.5%	
		Fully formula feeding	7	58.3%	5	41.%	
	Adolescents	Both	11	84.6%	2	15.4%	0.338
		Fully breastfeeding	8	61.5%	5	38.5%	
		Fully formula feeding	1	50%	1	50%	
Soft drink consumption	Early childhood	Daily	0	0.0%	1	100%	0.47
		Monthly	3	42.9%	4	57.1%٪	
		Never	21	77.8%	6	22.2%	
		Weekly	5	100%	0	0٪	
	School children	Daily	1	50%	1	50%	0.945
		Monthly	3	75%	1	25%	
		Never	16	_66.7%_	8	33.3%٪	
		Weekly	2	_66.7%_	1	33.3%	
	Adolescents	Monthly	3	_75%_	1	25%	0.985
		Never	12	70.6%	5	29.4%	
		Weekly	5	_71.4%_	2	28.6%	
		Daily	0	_0%_	0	0%	
Fast food consumption	Early childhood	Daily	1	100%	0	0%	0.821
		Monthly	2	66.7%	1	33.3%	
		Never	13	81.3%	3	18.8%	
		Weekly	13	65%	7	35%	
	School children	Daily	2	66.7%	1	33.3%	0.818
		Monthly	6	75%	2	25%	
		Never	4	50%	4	50%	
		Weekly	10	71.4%	4	28.6%	
	Adolescents	Daily	2	100%	0	0%	0.243
		Monthly	3	75%	1	25%	
		Never	5	100%	0	0%	
		Weekly	10	_58.8%_	7	41.2%	
Sleeping (9 p.m.)	Early childhood	After	24	77.4%	7	22.6%	0.196
		Before	5	55.6%	4	44.4%	
	School children	After	17	63%	10	37%	0.338
		Before	5	83.3%	1	16.7%	
	Adolescents	After	15	68.2%	7	31.8%	0.466
		Before	5	83.3%	1	16.7%	
Hours sleep/day	SD (range)	Early childhood	9,14±1.8		8.4±1.3		0.068
		School children	8.5±1.9		10.6±4.1		0.066
		Adolescents	9.3±2.1		8±1.5		0.142
Electronic devices use (minutes/day)	SD (range)	Early childhood	122± 92		185± 162		0.133
		School children	171± 117		185±130		0.771
		Adolescents	155± 122		291±290		0.32

**Figure 1:**
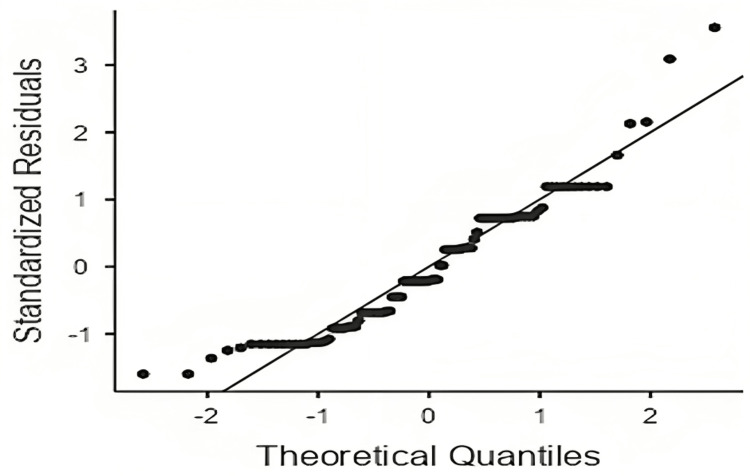
Q-Q plot screen time versus obesity

The average sleep duration was 9.03 hours for individuals with a normal BMI and 9.1 hours for those with a high BMI, yet this difference was not statistically significant. However, in early childhood, the mean sleep duration was 9.14±1.8 hours, and in school-aged children, it was 8.5±1.9 hours, which almost approached statistical significance in correlation with a normal BMI (p-value = 0.068, 0.066 respectively) (Tables 2, 3). Regarding electronic device usage, the average for those with a normal BMI was 147.61 ± 110.24 minutes, compared to 204.5 ± 164.76 minutes for those with a high BMI, where a significant association was found (p-value < 0.05), (Table 2). The Q-Q plot shows a normal distribution of the data, indicating an increased risk of high BMI with greater electronic device use (Figure 1). However, this significance dissipated when the data were categorized by age groups (Table 3).

## Discussion

Childhood obesity is a global health problem that has escalated over the last few decades. This study showed that the prevalence of overweight and obesity was almost one third (32.2% in boys and 26.2% in girls) of the studied children and adolescents, which is comparable with what has been published in the literature. 

The causes of obesity in children are multifactorial and include genetic, environmental, and biological factors. There has been an ongoing debate about whether there is a link between breastfeeding and childhood obesity, but it is inconclusive due to inconsistent evidence in the literature. This study shows no clear relationship between breastfeeding and obesity among children. Some studies, including a randomized controlled trial, have shown that there was no association between breastfeeding and childhood obesity [20]. In contrast, the American Academy of Pediatrics reported that there is a 20% decrease in the risk of obesity in breastfed children [16] In addition, a meta-analysis has shown that breastfeeding protects against childhood obesity [21]. Breast milk may be protective against obesity due to various potential biological mechanisms, but confounding factors can obscure this protective role of breastfeeding.

Surprisingly, the prevalence of exclusive breastfeeding (EBF) among the studied infants was 40.6%, which is notably higher compared to the prevalence rates of 25% or less found in many countries and the figure of 27.4% that was mentioned in a Saudi Arabian publication [14,15]. Despite these cases being compliant with the WHO definition of EBF, a previous publication from Saudi Arabia found that more than 90% of nursery children are exposed to CMF during the first days of life [22]. In addition, mothers often disregard small amounts of formula that their babies may receive, or, even if their babies had received formula for a short period, they may still consider the babies to be exclusively breastfed. This potential discrepancy in perception could explain the lack of a significant association between the type of feeding and childhood obesity in our study.

This study revealed a significant association between childhood obesity and electronic device usage, with more screen time being linked to a higher risk of obesity. This aligns with the findings of many studies; for example, a systematic review has shown that there is a strong association between screen time and adiposity in children and adolescents [23]. A meta-analysis has shown a positive correlation between childhood obesity and increased electronic device use [24]. However, another systematic review found inconsistent evidence of an association between screen time and obesity [25]. The probable association may be attributed to many reasons, such as sedentary behavior during screen time and screen time interfering with healthy behaviors such as exercise, physical activity, and eating nutritious meals.

Childhood obesity is a subject of debate in the literature, particularly regarding the causes, risk factors, and potential links to the persistent rise in the prevalence of obesity. The available research suggests that several factors could contribute to childhood obesity, including high birth weight, mothers smoking during pregnancy, pregnancy complications, fast food consumption, excessive screen time, and inadequate sleep. A systematic review has shown that high birth weight and mothers smoking during pregnancy are associated with later childhood overweight, while there is inconsistent evidence regarding screen time, feeding style, sleep time and duration [25]. Another systematic review has shown a positive relationship between childhood obesity and short sleep duration, but the findings of studies on screen time, sugar-sweetened beverages, and fat consumption are controversial [26]. The findings in this study show no statistically significant association with childhood obesity that could contribute to the controversy.

The rates of obesity found in Saudi studies are similar to what has been found in the United States and indicative of a lack of effective prevention programs to combat childhood obesity in Saudi Arabia. Preventing and managing childhood obesity requires a comprehensive approach that involves multiple stakeholders, including parents, healthcare professionals, schools, and policymakers. Many approaches have been implemented to decrease the prevalence of childhood obesity and control the persistent increase in the rate of obesity. Childhood obesity prevention protocols are predominantly school-based, but there are other prevention programs in various settings, including primary care settings, child care centers, and community centers [27, 28]. Unfortunately, none of these protocols have succeeded in reversing the increase in childhood obesity [27, 28]. The poorly understood impact of the complex factors that contribute to childhood obesity is the reason why it is challenging to comprehend how these factors affect children.

This study has several notable strengths, such as utilizing hospital records to ensure high-quality data and anthropometric measurements taken by a specialized pediatric nurse to ensure the accuracy of the data. However, it is essential to acknowledge certain limitations that may affect the interpretation of the findings. The study's sample size was relatively small, and it only included participants from a single city, which may restrict the generalizability of the findings to a wider population. The retrospective nature of this study and the wide age range of the subjects, there is a potential for significant recollection bias. In addition, the inability to quantify the precise amount of breastmilk in the 'mixed feeding' category makes it challenging to draw firm conclusions on the benefits of breastmilk exposure, as these benefits are known to be dose and duration-dependent. The data collection from a single hospital may constrain the external validity of the study findings. Lastly, the absence of follow-up measures that track changes in the weight of children throughout their development makes it difficult to establish a definitive causal relationship between breastfeeding and childhood obesity. Acknowledging these limitations is crucial for an understanding of the study's context and to guide future research.

## Conclusions

Obesity is a complex condition that arises from the interplay of biological, behavioral, social, environmental, and economic factors. The consistent rise in the rate of obesity is a major health concern. Risk factors for childhood obesity are still an area of contention, and the role of breastfeeding in this context remains contentious. The findings of the cross-sectional study aimed to contribute to the ongoing discussion on childhood obesity, yet they must be approached with caution owing to the small sample size and the broad age range of participants. The data suggests that there is no discernible relationship between breastfeeding and obesity. Moreover, there was an observable trend suggesting a possible association between higher screen time and increased rates of obesity and that sleep duration could potentially influence BMI in early childhood and school-aged groups. However, none of the other factors demonstrated a significant positive association with an increased risk of obesity. The confounding factors underscore the necessity for further research to elucidate the crucial role of breastfeeding in the prevention of childhood obesity. Addressing these factors may curb the escalating prevalence of childhood obesity and promote the health and well-being of children.
